# *In Silico* Prediction of Gamma-Aminobutyric Acid Type-A Receptors Using Novel Machine-Learning-Based SVM and GBDT Approaches

**DOI:** 10.1155/2016/2375268

**Published:** 2016-08-08

**Authors:** Zhijun Liao, Yong Huang, Xiaodong Yue, Huijuan Lu, Ping Xuan, Ying Ju

**Affiliations:** ^1^Department of Biochemistry and Molecular Biology, School of Basic Medical Sciences, Fujian Medical University, Fuzhou, Fujian 350122, China; ^2^College of Animal Science and Technology, Henan University of Science and Technology, Luoyang, Henan 471023, China; ^3^School of Computer Engineering and Science, Shanghai University, Shanghai 200444, China; ^4^College of Information Engineering, China Jiliang University, Hangzhou, Zhejiang 310018, China; ^5^School of Computer Science and Technology, Heilongjiang University, Harbin, Heilongjiang 150080, China; ^6^School of Information Science and Technology, Xiamen University, Xiamen, Fujian 361005, China

## Abstract

Gamma-aminobutyric acid type-A receptors (GABA_A_Rs) belong to multisubunit membrane spanning ligand-gated ion channels (LGICs) which act as the principal mediators of rapid inhibitory synaptic transmission in the human brain. Therefore, the category prediction of GABA_A_Rs just from the protein amino acid sequence would be very helpful for the recognition and research of novel receptors. Based on the proteins' physicochemical properties, amino acids composition and position, a GABA_A_R classifier was first constructed using a 188-dimensional (188D) algorithm at 90% cd-hit identity and compared with pseudo-amino acid composition (PseAAC) and ProtrWeb web-based algorithms for human GABA_A_R proteins. Then, four classifiers including gradient boosting decision tree (GBDT), random forest (RF), a library for support vector machine (libSVM), and k-nearest neighbor (*k*-NN) were compared on the dataset at cd-hit 40% low identity. This work obtained the highest correctly classified rate at 96.8% and the highest specificity at 99.29%. But the values of sensitivity, accuracy, and Matthew's correlation coefficient were a little lower than those of PseAAC and ProtrWeb; GBDT and libSVM can make a little better performance than RF and *k*-NN at the second dataset. In conclusion, a GABA_A_R classifier was successfully constructed using only the protein sequence information.

## 1. Introduction

**Figure 1:**
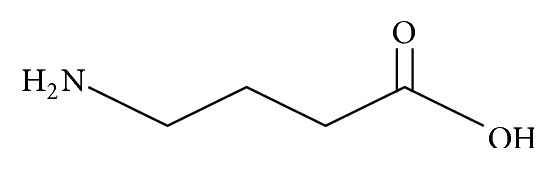
GABA conformation.

**Figure 2:**
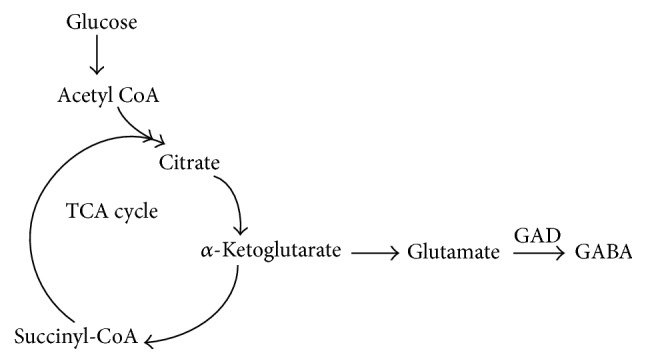
Model of direct GABA production.

Gamma-aminobutyric acid (GABA) is a major human brain inhibitory neurotransmitter and plays a principal role in the regulation of pituitary gland function. GABA is made up of a four-carbon chain flexible carbon skeleton (Figure 1), which can adopt a number of conformations when interacting with many macromolecular receptor targets. This characteristic of GABA can provide many selective ligands by producing conformationally restricted analogues [1]. GABA is mainly synthesized in the hypothalamus as well as within the pituitary gland and stored in the anterior lobe and intermediate lobe cells, the GABA-synthesizing enzyme is glutamic acid decarboxylase (GAD) which is relevant to TCA cycle [2], and the direct substrate is glutamate [3] (Figure 2). In addition to GAD, the GABA level is also related to glutamine-glutamate (Gln-Glu) cycling [4], in which glutaminase and glutamine synthetase play a key role in keeping the cycling balance. Gln is first converted to Glu and then to GABA in the cycle, or Glu solution is catalyzed to GABA; this process is known to play a significant role in the regulation of neurogenesis, and the release of GABA is mainly produced from Purkinje cells in the cerebellar cortex via special regulatory mechanism [5–7].

**Figure 3:**
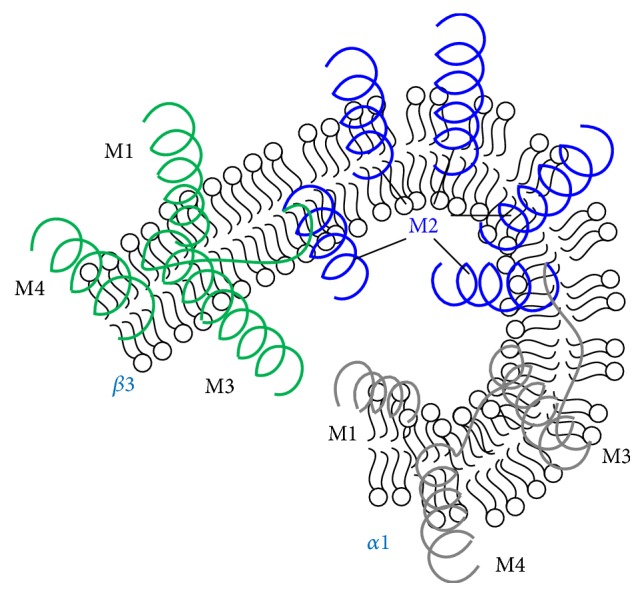
GABA_A_R modulation patterns of transmembrane domain, a homology model of the transmembrane domains of a GABA_A_R showing the five-M2-helix domains forming the chloride ion channel (blue) and M1, M3, and M4 helices for single *α*1 (grey) or *β*3 (green) subunit. The helices may embed into the postsynaptic membrane in mammalian CNS.

GABA can specifically interact with the postsynaptic GABA receptor in human central nervous system (CNS) [8]; the specific binding of GABA to synaptic membrane fractions is saturable. Three types of GABA receptors are expressed in human, namely, the ionotropic GABA_A_ receptor (GABA_A_R), the metabotropic GABA_B_ receptor (such as G protein-coupled receptor) [9], and another ionotropic GABA_C_ receptor, among them GABA_A_R is relevant to epilepsy [10]. These receptors belong to the Cys-loop superfamily of ligand-gated ion channels (LGICs) and exhibit a long (about 200 a.a.) extracellular amino terminus, which is thought to be responsible for ligand channel interactions. The amino terminus forms agonist or antagonist binding sites, four transmembrane (TM) domains, and a large intracellular domain between TM3 and TM4 for phosphorylating regulation and localization at synapses, and five TM2 domains in a cycle form the lining segment of the ion channel (Figure 3). The extracellular amino terminus contains a conserved motif, called the Cys-loop (13-amino acid disulfide loop), which is characterized by 2 cysteine residues spaced by 13 different amino acid residues [11]; the amino terminus incorporates neurotransmitters and some modulator binding sites. For example, the extracellular domain of GABA_A_R  *β*2 subunits contains the amino acid residue “CMMDLRRYPLDEQNC” (C stands for cysteine). For the structural details of Cys-loop receptors see review [12].

GABA_A_Rs form pentameric chloride channels comprising various combinations from eight kinds of subunits (*α*, *β*, *γ*, *δ*, *ε*, *θ*, *π*, and *ρ*), each of which comprises several subtypes [13]. These receptors belong to a superfamily of pentameric ligand-gated ion channels (pLGICs) with five-membered ring structures; pLGICs are also known as Cys-loop receptors including two classes: the cation-selective (e.g., nicotinic acetylcholine receptors and serotonin type 3 receptors) and anion-selective (e.g., glycine receptors (GlyRs) and GABA_A_Rs) [14]. According to their extracellular domain, pentameric receptors can be further divided into these containing only one conserved Cys-loop and those containing an additional disulfide bond that links the *β*9-*β*10 strands in Loop C. Human GABA_A_R subunits are encoded by 19 different genes, namely, *α*1–6, *β*1–3, *γ*1–3, *δ*, *ε*, *θ*, *π*, and *ρ*1–3; among these subunits, the crystallization shows that human GABA_A_R  *β*3 subunit is unique to eukaryotic Cys-loop receptors [15]. The *α*1–*α*6 subunits are encoded by* GABRA1 *to* GABRA6* genes; the *α*1 subtype is widely expressed in the whole brain, whereas *α*2, *α*3, *α*4, *α*5, and *α*6 subtypes are expressed in specific brain areas [16]. Most of the pentameric GABA_A_Rs in the human brain are typically composed of two *α* subunits, two *β* subunits, and one *γ* subunit, and the GABA binding sites are located in the *α*-*β* subunit interface [17]. The *α*1, *β*2, and *γ*2 subunits are expressed most abundantly in human brain [18], and the subunit variants may thus influence ion channel gating, expression, and GABA receptor trafficking to the cell surface. The* GABRA1 *and* GABRA6 *genes are located in human chromosome 5, whereas* GABRA2 *and* GABRA3* are located in chromosome 4 and* GABRA4 *and* GABRA5 *are located in chromosome X and chromosome 15, respectively [19]. These genes have been proposed to affect certain drug targets and the regulation of neuronal activities in human brain [20]. Several antiepileptic drugs (AEDs) such as phenobarbital and gabapentin bind to GABA_A_Rs in the CNS with a confined area distribution, and the alterations in GABA_A_R subunits may regulate the responses elicited by AEDs [21]. Several AEDs exert agonistic effects on GABA_A_Rs. AEDs may react with GABA_A_Rs comprising distinct subunits in diverse manners, and the composition and function of *α* subunits may influence the treatment efficacy of different AEDs [22]. Targeted proteins of AEDs are involved in the regulation of extracellular K^+^ and intracellular Cl^−^ homeostasis, cell volume, and pH, all of which are important for maintaining normal brain activity [23].

GABA_A_R subunit mutations or genetic variations can lead to its dysfunctions, which have been thought to participate in the pathomechanisms of epilepsy [24], in which multiple GABA_A_R epilepsy mutations result in protein misfolding and may cause degradation or retention of the protein molecules in cells; Kang et al. found that mutant GABA_A_R  *γ*2 subunits accumulate and aggregate intracellularly, activated caspase-3, and caused widespread and age-dependent neurodegeneration; these findings suggested the epilepsy-associated mutant *γ*2 subunit played important role in neurodegeneration [25]. The gene mutations or genetic variation of the *α*1, *α*6, *β*2, *β*3, *γ*2, or *δ* subunits (GABRA1, GABRA6, GABRB2, GABRB3, GABRG2, and GABRD, resp.) compromises hyperpolarization through GABA_A_Rs, and these variations have been associated with human epilepsy with or without febrile seizures [26].

Support vector machine (SVM) is a kind of supervised machine learning algorithms that have been broadly applied for classification and regression analysis [27–32], which is also a type of sparse kernel machines that rely on various data to predict unknown class labels and which has linear or nonlinear learning model for binary classifier [33–35]. Random forest (RF) is an ensemble machine learning technique based on random decision trees for classification and other tasks. Relying on the feature, a data point can be divided into a special category and is assigned a prediction. RF has been broadly applied in novel protein and target identification [36, 37], because it combines the merits of bagging idea and feature selection [38]. Another decision tree learning is gradient boosting decision tree (GBDT), which has been very successfully applied for many fields such as smart city concept [39], and its major advantage is ability to find nonlinear interactions automatically through decision tree learning with the minimality error. GBDT is generally regarded as one of the best out-of-the-box classifiers which has the ability to generalize and can combine weak learners into a single strong learner; it has gradually acquired popularity in the field of machine learning methods although it still possesses many disadvantages [40–43].

Here, we performed an* in silico* analysis on the GABA_A_Rs according to sequence information and other physicochemical features, including hydrophobicity, normalized van der Waals volume, polarity, polarizability, charge, surface tension, secondary structure, and solvent accessibility. Twenty natural amino acids can be divided into 3 different groups based on each of the above eight properties, and thus 188-dimensional (188D) feature vectors of proteins were constructed with an ensemble classifier [44], which performed well in membrane protein prediction [45]. We employed PseAAC and ProtrWeb methods for human GABA_A_R to adapt to the web server limit of sequence amounts; we also applied libSVM, RF, GBDT, and widely used *k*-nearest neighbor (*k*-NN) algorithms to make comparisons of performance with dataset at rigorous cd-hit filtration [46].

Since* motif*, a conserved short pattern of a protein [47], is one of the fundamental function units of molecular evolution, with regard to DNA, a motif may act as a protein-binding site; in proteins, a motif may directly correspond to the active site of an enzyme or a structural unit of the protein. Therefore, we also conducted motif analysis.

## 2. Materials and Methods

### 2.1. Data Retrieval and Treatment

All the primary sequences of both GABA_A_R and the control Pfam proteins (in FASTA files) were retrieved from the UniProt database (http://www.uniprot.org/); the raw data are preprocessed by cd-hit program (http://cd-hit.org) to merge the sequence similarities and reduce the complexity [46]. To avoid bias in the classifier, we set the identity at 90% similarity and obtained the results of 2353 GABA_A_R sequences as positive dataset; the negative samples were obtained from the control proteins when the positive ones were deleted, and 10652 entries were obtained as negative dataset. When the four classifiers performance was measured, cd-hit was set at rigorous 40% identity and gained 360 GABA_A_Rs and 9598 non-GABA_A_Rs.

### 2.2. Prediction Analysis for Potential GABA_A_R Proteins

Machine learning is often employed in the bioinformatics and proteomics problem. Several important techniques facilitate the protein classification and identification, such as imbalanced classification strategies [48], ensemble learning [49–51], samples selection strategies [52, 53], features reduction, and ranking methods [54–56].

To predict the potential GABA_A_R from the amino acid sequences, we constructed a classifier according to the GABA_A_R protein features. First, we extracted the feature vectors from positive versus negative protein sequence dataset by using a novel machine-learning-based method developed by our group, we transformed all the positive and negative sequences into the corresponding protein family (Pfam) information, and the obtained features included sequence evolutional information, *k*-skip-*n*-gram model, physicochemical properties, and local PsePSSM [57]. Altogether, we assembled 188D feature vectors. Afterward, the resulting feature vectors were imported into Weka (http://www.cs.waikato.ac.nz/ml/weka/), which is a machine learning workbench used for automatic classification via visualization and cross-validation analysis [58, 59]. After several preliminary trials with the same dataset, we selected random forest method and set the parameters as default.

### 2.3. Conserved Motif Analysis of Human GABA_A_R Proteins

Conserved motif analyses were implemented using the online MEME Suite (http://meme-suite.org/, 4.11.1 version), a powerful motif-based sequence analysis tool, which integrated a set of web-based tools including Gene Ontology database for studying sequence motifs in proteins, DNA, and RNA [60]. Currently, the MEME Suite has added six new tools and reached thirteen since the “Nucleic Acids Research” Web Server Issue in 2009. Human GABA_A_R sequences in FASTA format were used as a file input. The maximum motif width, minimal motif width, and maximum number of motifs were set to 50, 6, and 9, respectively. The remaining parameters were set as default values.

### 2.4. Pseudo-Amino Acid Composition and ProtrWeb Analysis

Chou et al. [61–63] had proposed the concept of PseAAC to describe global or long-range sequence-order protein information early in 2001; their original design objective was to improve protein subcellular localization prediction and membrane protein type prediction. Since then, the PseAAC approach alone or incorporating other properties had rapidly penetrated many areas of computational proteomics. As the most intuitive features for protein biochemical reactions, the physicochemical properties of amino acids significantly influence the protein classification. Features that incorporate appropriate physicochemical properties can contain much valuable information for improving the performance of predictors. Single feature extraction of our own method has inevitably its own shortcomings and does not always perform well on all circumstances. Thus, we also used the concept of PseAAC and ProtrWeb (http://protrweb.scbdd.com/) to construct feature vectors for human GABA_A_R proteins (58 entries) and other proteins (58 entries) in this study.

PseAAC is a web server that can generate numerous pseudo-amino acid compositions including sequence-order information in addition to the conventional 20D amino acid composition. It is a classification algorithm based on the amino acid composition and physicochemical characteristics of proteins; the server was designed in a flexible way to identify various pseudo-amino acid composition information for a given protein sequence by selecting different parameters and their combinations. PseAAC provides three PseAA modes and six amino acid characters for user to choose. ProtrWeb [64] is also a web server based on the R package routine protr, the first version of which was developed in November 2013. This server is dedicated to calculate protein sequence-derived structural and physicochemical descriptors such as amino acid composition. *n*-gram and *k*-skip are based on permutation and combination theory. ProtrWeb can be applied in various protein prediction studies, including protein structural and functional classes, protein subcellular locations, protein-protein interactions, and receptor-ligand interactions. ProtrWeb offers 12 types of commonly used descriptors presented in the web such as amino acid composition, dipeptide composition, and pseudo-amino acid composition. Recently, some studies have shown that the long-range sequence-order effects of DNA [65] can improve the performance of computational predictors [66].

To extract features from the physicochemical properties of proteins by using PseAAC, we considered all six physiochemical properties: hydrophobicity, hydrophilicity, mass, pK1 (alpha-COOH), pK2 (NH3), and pI (at 25°C). We selected type 2 PseAA mode, set Lambda parameter at 10, and set the weight factor as default. The results were shown as 80-dimensional (80D) data for each protein. For ProtrWeb, we chose amino acid composition (20 Dim) and pseudo-amino acid composition (50 Dim) adapted to the restricted parameter measure.

### 2.5. Prediction Ability Comparison of Four Classifiers on the 40% Identity cd-Hit Filtration Data

We extracted 188D feature vectors from 360 GABA_A_Rs and 9598 non-GABA_A_Rs as input to Weka performing category via RF, *k*-NN, and SVM algorithm which was implemented using libSVM. GBDT classifier was carried out by python program developed by ourselves; the above 4 classifiers have the parameters set as default.

Four common measurements were used to illuminate the performance quality of the predictor more intuitively. Sensitivity (Sn), specificity (Sp), accuracy (Acc), and Matthew's correlation coefficient (MCC) were adopted to evaluate the above three methods and four classifiers. These methods are formulated as follows:

(1) Sn=TPTP+FN,Sp=TNTN+FP,Acc=TP+TNTP+FP+TN+FN,MCC=TP∗TN−FP∗FNTP+FNTP+FPTN+FPTN+FN,

where TP, TN, FP, and FN stand for the numbers of true positive, true negative, false positive, and false negative, respectively.

## 3. Results

### 3.1. Searching the Protein Family Number

To determine the Pfam families of GABA_A_Rs, we ran the program with the positive and negative protein sequences (GABA_A_Rs versus non-GABA_A_Rs) and obtained nonredundant Pfam numbers after combining the same ones (Table 1). The negative group was very large; thus, we only listed the positive ones.

### 3.2. Reclassification of Positive and Negative Proteins

**Figure 4:**
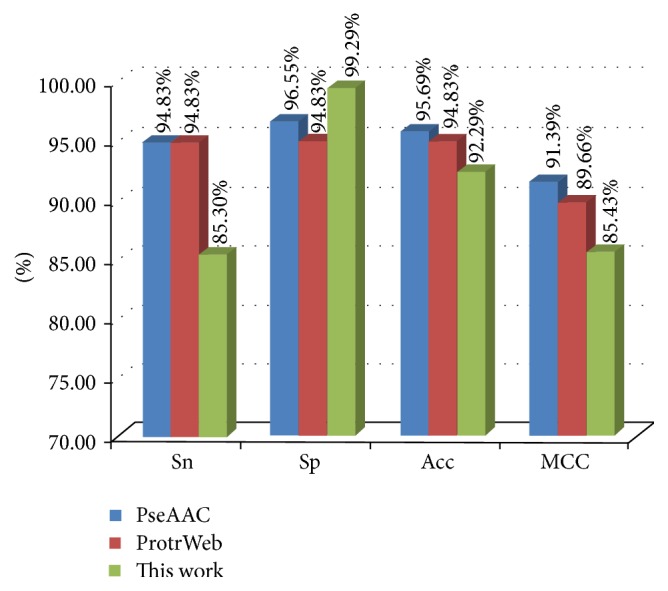
Sn, Sp, Acc, and MCC values listed from PseAAC, ProtrWeb, and our work. Note: PseAAC and ProtrWeb only include human 58 GABA_A_Rs and 58 non-GABA_A_Rs because of the web amount limitation; our method contains all the GABA_A_Rs and non-GABA_A_Rs (2353 versus 10652).

We obtained the 188D (this work), 80D (from PseAAC), and 70D (from ProtrWeb) feature vector dataset from both positive and negative groups and used them as input to the Weka explorer (RF algorithm). The results showed that the correctly classified rates were 96.8%, 95.7%, and 94.8%. The confusion matrix is shown in Table 2, and the four common measurement values are illustrated in Figure 4.

### 3.3. Four Classifiers' Prediction Ability Comparison

On the four classifiers, they all performed well and got high correctly classified rate over 96%, but GBDT and libSVM had a little better performance than RF and *k*-NN assessed from all the indicators (Table 3).

### 3.4. Conserved Motif Analysis of Human GABA_A_R

**Figure 5:**
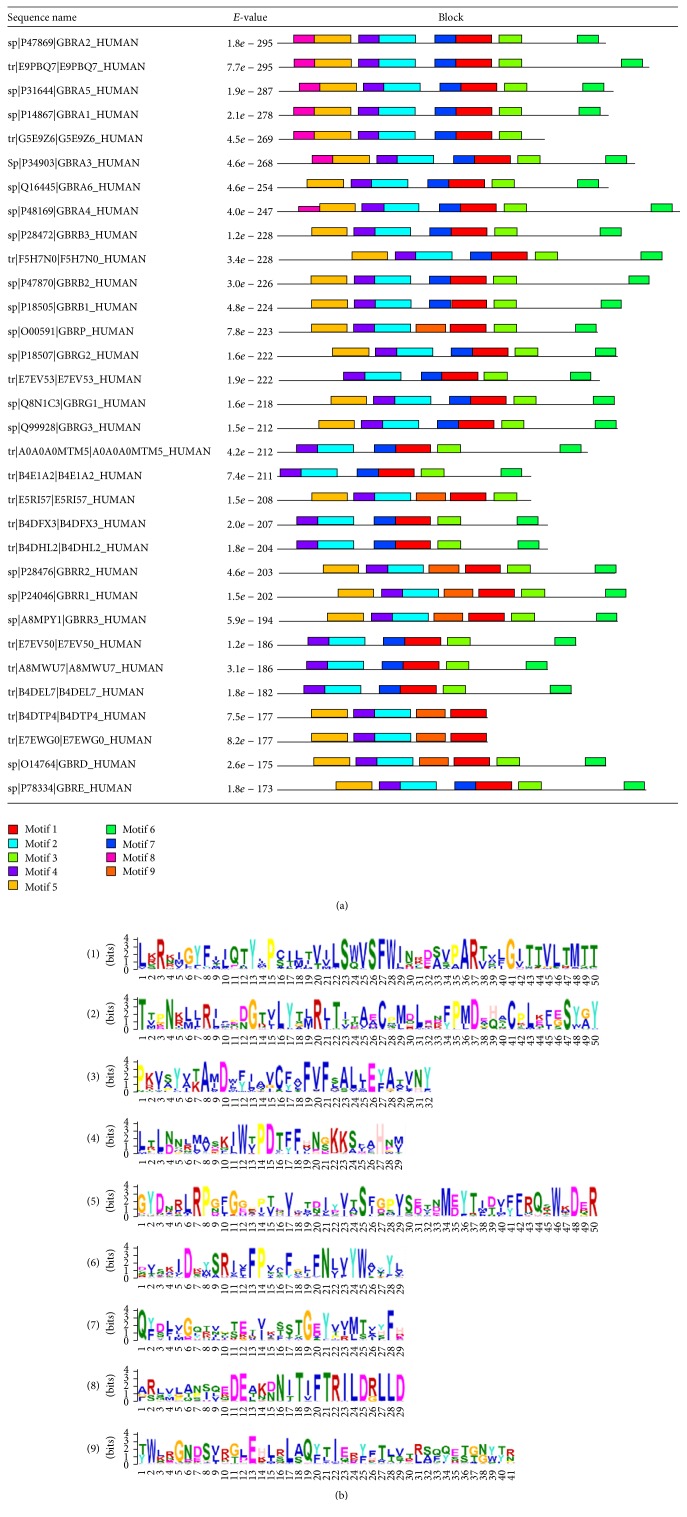
Motifs of human GABA_A_Rs found by the MEME system (for details see Table 3). (a) Locations of the nine discovered motifs (showing the top 32 sequences). (b) Nine motif logos found by MEME.

To reveal the evolutionary correlation of GABA_A_Rs from the conserved motifs, 92 human protein sequences were analyzed by using MEME software. The nine most significant and conserved motifs are shown in Figure 5 and Table 4.

## 4. Discussion

The primary structures of amino acid sequences are often the basis for understanding the three-dimensional conformation and functional properties of proteins [67], which exhibit an intimate relationship between their primary structure and function [68]. Twenty natural *α*-amino acids commonly constitute the primary sequences of proteins [69, 70]. Amino acids are biologically important organic nitrogenous compounds in the natural world. These compounds contain amine (-NH_2_) and carboxylic acid (-COOH) functional groups which link with the same carbon atom called *α*-carbon, usually along with a side-chain (called R group) specific to each amino acid. The elements of carbon, hydrogen, oxygen, and nitrogen are essential for an amino acid, though other elements are found in the R group. Amino acids can be classified in many ways, such as according to the core structure and side-chain group properties. However, 20 standard and encoding *α*-carbon amino acids are usually classified into five main groups on the basis of biochemistry [71], namely, a hydrophobe, if the side-chain is nonpolar; a hydrophile, if it is polar but uncharged; aromatic, if it includes an aromatic ring; acidic, if it is negatively charged; and basic, if it is positively charged.

Previous research has extracted information on protein feature according to composition, position, or physicochemical properties [31]. In our work, we adopted 188D algorithm to extract feature vectors by combining amino acid compositions with physicochemical properties in a protein functional classifier [72]. This 188D method includes amino acid composition (20D) and eight types of physicochemical properties, that is, hydrophobicity (21D), normalized van der Waals volume (21D), polarity (21D), polarizability (21D), charge (21D), surface tension (21D), secondary structure (21D), and solvent accessibility (21D). The CTD model was employed to describe global information about the protein sequence, where C represents the percentage of each type of hydrophobic amino acid in an amino acid sequence, T represents the frequency of one hydrophobic amino acid followed by another amino acid with different hydrophobic properties, and D represents the first, 25%, 50%, 75%, and last position of the amino acids that satisfy certain properties in the sequence; for details, see [44]. In addition to this 188D feature vector extraction method, we used two web-based servers, PseAAC and ProtrWeb, for 80D and 70D feature vectors, respectively. The limited amount of sequence on the web allowed the analysis of only human GABA_A_Rs and the corresponding non-GABA_A_Rs by using the last two methods.

The abnormities of GABA_A_Rs are associated with the pathology and progression of several neurological and psychiatric diseases, such as autism, schizophrenia [73], and alcoholism [74], particularly in epilepsy [75–79], Dravet syndrome [80], asthma [81], breast cancer [82], some psychiatric diseases [83], Alzheimer disease [84], and other neurodegenerative diseases. It is recently reported that GABA_A_R may be involved in apoptosis in preeclampsia [85]. Human GABA_A_Rs conserved motifs analyses indicate that motifs 1, 3, and 6 are the frame of neurotransmitter-gated ion channel transmembrane region, which form the ion channel for cation transporter by the construction of transmembrane helix. Motifs 2, 4, and 5 are also composed of neurotransmitter-gated ion channel extracellular ligand binding domain by linking closely and forming a pentameric arrangement in the structure [86]. Various GABA receptor genes are associated with many mental-disorder-related phenotypes. Alterations in GABAergic inhibitory actions, such as the subunit amount, composition, and gene expression of GABA_A_Rs, may demonstrate neurophysiologic and functional consequences related to mental disorders. Some studies on protein prediction using Chou's method have been reported in 2011 because of the importance of GABA_A_Rs [11]. However, similar studies on GABA_A_Rs are rarely reported since then.

The current results showed that our method reached the most correctly classified instances at 96.8%; it suggested that our 188D algorithm performed well for classification and could correctly discriminate both positive and negative samples with relative high specificity. However, the Sn, Acc, and MCC indexes were lower than those of the PseAAC and ProtrWeb methods; this might be due to the large dataset size of our work. But the lowest value was higher than 85%. Overall, our project, which is mainly based on physicochemical properties, can reflect the characteristics of protein sequences and can be applied in the prediction of GABA_A_Rs classification. Definitely, it needs to develop more precise methods based on 188D.

## Figures and Tables

**Table 1:** Pfam accession numbers (83 entries) for GABA_A_Rs as positive group.

PF00008, PF00012, PF00018, PF00022, PF00028, PF00053, PF00055, PF00057, PF00059, PF00060, PF00069
PF00078, PF00084, PF00087, PF00090, PF00100, PF00130, PF00147, PF00163, PF00168, PF00169, PF00209
PF00226, PF00240, PF00270, PF00271, PF00335, PF00387, PF00388, PF00397, PF00400, PF00454, PF00520
PF00564, PF00621, PF00627, PF00643, PF00651, PF00665, PF00754, PF00850, PF00892, PF01082, PF01352
PF01436, PF01479, PF01498, PF01529, PF02072, PF02140, PF02214, PF02259, PF02260, PF02460, PF02891
PF02931, PF02932, PF02991, PF03144, PF03416, PF03521, PF04849, PF06220, PF07645, PF07690, PF07707
PF08007, PF08266, PF08377, PF08625, PF08771, PF09279, PF09497, PF11865, PF11938, PF12248, PF12448
PF12662, PF13499, PF15311, PF15974, PF16457, PF16492

**Table 2:** Confusion matrix classifier (RF) from three kinds of feature vector extraction algorithms.

	PseAAC		ProtrWeb		This work	
	Human GABA_A_Rs	Human non-GABA_A_Rs	Human GABA_A_Rs	Human non-GABA_A_Rs	GABA_A_R proteins	Non-GABA_A_R proteins
Positive cases	55	2	55	3	2007	76
Negative cases	3	56	3	55	346	10576

**Table 3:** Classification results for four classifiers based on 360 GABA_A_Rs and 9598 non-GABA_A_Rs.

Classifier	Sensitivity (%)	Specificity (%)	Accuracy (%)	MCC	Correctly classified rate
GDBT	51.39	99.66	75.52	0.5828	0.9791
RF	41.39	99.86	70.63	0.5085	0.9775
libSVM	58.89	97.76	78.32	0.6148	0.9635
*k*-NN	51.94	98.17	75.06	0.5651	0.9650

**Table 4:** Human conserved motifs of GABA_A_Rs found by MEME system (in regular expression).

Motif	Width	*E*-value	Best possible match
1	50	1.6*e* − 1592	L[KRS]R[KNR][IMV]GYF[IV][IL]QTY[IL]P[CS][IT][LM][TI][VT][IV]LS [WQ]VSFW[IL]N[RK][DE][SA][VS][PA]AR[TV][VAS][LF]G[IV] TTVLTMTT

2	50	8.6*e* − 1477	T[TV]PN[KR][LM][LI]R[IL]F[PD][DN]GT[VLI]LYT[LM]R[LI]T[ITV]TA [EA]C[PN][ML][DQ]L[ES][DNR][FY]P[ML]D[EAT][HQ][AST]CPL[KE] [FL][EG]SY[GA]Y

3	32	1.9*e* − 816	P[KR][VI][SA]Y[VAI][TK]A[MI]DW[FY][IL]AVC[FY][AV]FVF[SL]AL [LI]E[YF]A[TA][VL]NY

4	29	2.4*e* − 804	L[TR]L[ND]N[LR][ML][AV]SK[IL]W[TV]PDT[FY]F[HRV]N[GS]KKS[FIV] AHN[MV]

5	50	3.7*e* − 1083	GYDNRLRP[GN][FL]G[GE][PR][PI][TV][EQ][VI]XT[DN]I[YD][VI][TA] S[FI][GD][PS][VI]S[DE][TV][ND]M[ED]YTI[DT][VI][FY][FL]RQ [SKT]WKDER

6	29	1.7*e* − 538	[DS][VI]S[KA]ID[KR][YW]SRI[VFL]FPV[AL]FG[LF]FN[LV]VYW[AVL] [YTV]Y[LV]

7	29	3.5*e* − 413	Q[FY][DS][LFI][VL]G[QL][TR][VN][GST][TS]E[TI][VI]K[STF]STG[ED] Y[VPT][VIR][ML][TS][VLA][YHS]FH

8	29	2.4*e* − 262	[AFG][RS][LQS][VMY][LGP][AQT][NPS][IS][QLV][EKQ]DE[ALT][KN] [DN]N[IT]T[IV]FTRILD[RG]LLD

9	41	5.8*e* − 174	[TY]W[LK]RGN[DE]S[VL][RK][GT][LD]E[HK][LI][RS]L[AS]Q[YF][TL] I[EQ]R[YF][FH]T[LT][VS]T[RL][SA][QF][QY][ES][TS][GT][NG][YW] [TY][RN]
